# Supplementary material for: Selection Signatures in Italian Livestock Guardian and Herding Shepherd Dogs
Source: Vet Sci. 2022 Dec 21;10(1):3. doi: 10.3390/vetsci10010003 (PMC9862740; doi:10.3390/vetsci10010003)
Supplement: Supplementary file 1 [file vetsci-10-00003-s001.zip › supplementary/Table S1.pdf]

Table S1. Dog breeds included in the present study.

Breed name	Breed symbol	Group	Initial dataset (n.)	Quality check (n.)*	Size reduction (n.)**
**Pastore Apuano**	APUA	Herding dogs (HD)	19	18	18
**Bergamasco shepherd dog**	BERG	Herding dogs (HD)	15	11	11
**Pastore d'Oropa**	DORO	Herding dogs (HD)	15	15	15
**Lupino del Gigante**	LUGI	Herding dogs (HD)	23	18	18
**Pastore della Lessinia e del Lagorai**	PALA	Herding dogs (HD)	10	10	10
**Fonni's dog**	FONN	Livestock guardians (LGD)	30	30	24
**Mannara dog**	MANN	Livestock guardians (LGD)	12	12	12
**Maremma and the Abruzzi sheepdog**	MARM	Livestock guardians (LGD)	20	16	16
**Pastore della Sila**	SILA	Livestock guardians (LGD)	14	14	14

*This dataset was used for population structure analyses (multidimensional scaling, genetic distances, and Admixture).
**This dataset was used for selection signature analyses (XP-EHH, F_{ST}, and ROH).
