# Supplementary material for: Selection Signatures in Italian Livestock Guardian and Herding Shepherd Dogs
Source: Vet Sci. 2022 Dec 21;10(1):3. doi: 10.3390/vetsci10010003 (PMC9862740; doi:10.3390/vetsci10010003)
Supplement: Supplementary file 1 [file vetsci-10-00003-s001.zip › supplementary/Table S2.pdf]

Table S2. Mean Q-score of each of the seven clusters identified in the admixture analysis. The clusters were called after the breed they represented the most.

	**MARM cluster**	**BERG cluster**	**FONN cluster**	**APUA cluster**	**LUGI cluster**	**DORO cluster**	**SILA cluster**
**APUA**	5.2%	6.1%	9.7%	47.9%	24.6%	4.5%	2.1%
**BERG**	3.0%	80.2%	5.1%	3.5%	4.1%	2.5%	1.5%
**DORO**	4.0%	3.9%	5.2%	3.1%	6.3%	76.5%	1.1%
**FONN**	12.8%	5.0%	69.3%	4.4%	3.0%	2.4%	\3.1%
**LUGI**	2.5%	3.3%	4.3%	8.2%	77.3%	3.1%	1.2%
**MANN**	42.5%	7.4%	23.8%	11.4%	5.4%	3.8%	5.7%
**MARM**	92.9%	1.1%	2.8%	1.0%	0.4%	0.8%	1.1%
**PALA**	13.3%	10.5%	17.2%	18.9%	25.5%	9.6%	5.0%
**SILA**	13.1%	2.6%	7.4%	3.0%	1.5%	1.9%	70.4%

Herding shepherd dog breeds: Pastore Apuano (APUA), Bergamasco shepherd dog (BERG), Pastore d'Oropa (DORO), Lupino del Gigante (LUGI), and Pastore della Lessinia e del Lagorai (PALA); livestock guardian breeds: Fonni's dog (FONN), Mannara dog (MANN), Maremma and the Abruzzi sheepdog (MARM), and Pastore della Sila (SILA).
